# From Food Contaminant to Therapeutic Target: Identification of KCNE2 and 5-Azacytidine for Gastric Cancer via Multi-Omics, Machine Learning, and In Vitro Validation

**DOI:** 10.3390/ph19071060

**Published:** 2026-07-09

**Authors:** Meimei Chen, Shaohua Zheng, Tingjian Wu, Jiaqi Wu, Ruina Huang, Zhaoyang Yang, Huijuan Gan

**Affiliations:** 1 College of Traditional Chinese Medicine, Fujian University of Traditional Chinese Medicine, Fuzhou 350122, China; chenmeimei1984@163.com (M.C.);; 2 Fujian Key Laboratory of TCM Health Status Identification, Fujian University of Traditional Chinese Medicine, Fuzhou 350122, China; 3 College of Physics and Information Engineering, Fuzhou University, Fuzhou 350108, China

**Keywords:** Benzo[a]pyrene, gastric cancer, machine learning algorithms, WGCNA, risk model, molecular dynamics, in vitro validation

## Abstract

**Background:** Benzo[a]pyrene (BaP), a common food contaminant, is a recognized gastric carcinogen. This study aimed to identify therapeutic targets and repurposed drugs for gastric cancer (GC) using BaP as a network toxicology query. **Methods:** An integrated strategy combining network toxicology, multi-omics, machine learning (Random Forest, LASSO, SVM-RFE), and experimental validation was applied. **Results:** By intersecting GC-associated genes with BaP-related targets and machine learning, we identified three hub genes. The logistic regression model further revealed KCNE2 as a protective factor (OR = 0.515, 95% CI: 0.383–0.692), while SULF1 (OR = 2.940, 95% CI: 1.399–6.179) and TIMP1 (OR = 5.351, 95% CI: 2.020–16.743) were identified as potential risk factors. Survival analysis confirmed their prognostic significance. Single-cell transcriptomics descriptively showed TIMP1 and SULF1 enrichment in malignant/stromal cells and fibroblasts, respectively, whereas KCNE2 was restricted to normal epithelial cells and silenced in tumors. GSVA implicated epigenetic regulation, ECM remodeling, and TGF-β signaling. Molecular docking and dynamics simulations suggested that BaP can form stable complexes with DNMT1 and DNMT3A. Accordingly, drug enrichment analysis identified DNMT inhibitor 5-azacytidine as a top candidate. Cellular experiments confirmed that 5-azacytidine selectively inhibited GC cells and was associated with modulation of the DNMT3A–KCNE2 axis. **Conclusions:** Our findings provide a novel molecular target and a repurposed drug for GC from the perspective of a food contaminant.

## 1. Introduction

Benzo[a]pyrene (BaP) is a prototypical polycyclic aromatic hydrocarbon (PAH) that has been classified as a Group I carcinogen by the International Agency for Research on Cancer (IARC), indicating its unequivocal carcinogenicity in humans. Its ubiquitous presence in human environments (such as incompletely combusted organic matter and high-temperature cooked foods like fried items and barbecues) has attracted considerable attention [1]. Dietary intake is a primary route of human exposure to BaP. The stomach, as the first digestive organ encountered, is a direct target of this exposure. Early animal experiments have demonstrated that oral administration of BaP can induce gastric tumors, such as squamous cell carcinoma and papilloma, in mouse models [2]. The classical carcinogenic mechanism posits that BaP itself acts as a procarcinogen, requiring metabolic activation in the body via cytochrome P450 enzyme systems (such as CYP1A1 and CYP1B1) to form highly reactive epoxides (like BPDE). This metabolite can covalently bind to DNA, resulting in the formation of DNA adducts that subsequently provoke gene mutations and ultimately lead to cellular carcinogenesis [3,4]. Furthermore, research indicates that the toxic effects of BaP are closely associated with the activation of the aryl hydrocarbon receptor (AhR) signaling pathway [5]. The activation of AhR not only regulates the expression of metabolic enzymes but may also induce the expression of genes such as MMP9 and c-myc through the downstream ERK signaling pathway, thereby facilitating the proliferation and invasion of GC cells [6]. Although these foundational studies have revealed certain aspects of BaP’s carcinogenicity, the precise regulatory mechanisms within its molecular network, particularly the key driver genes and signaling pathways, still remain unclear. Given that environmental carcinogens often perturb fundamental oncogenic pathways, elucidating the BaP-related molecular network offers a powerful strategy to uncover critical vulnerabilities in GC.

In recent years, network toxicology has enabled rapid prediction of potential toxic targets and signaling pathways of chemical substances at the molecular level by constructing and analyzing complex biological molecular networks composed of nodes such as “compounds–targets–genes–diseases”. This approach provides an efficient and comprehensive perspective for risk assessment and mechanistic exploration of environmental pollutants. Numerous studies have demonstrated that network toxicology plays a prominent role in research on environment-related diseases, not only uncovering many previously unknown disease mechanisms but also precisely identifying potential therapeutic targets [7,8]. Moreover, Weighted Gene Co-expression Network Analysis (WGCNA) is a powerful systems biology method that can classify thousands of genes into several highly co-expressed “modules” after confirming that the gene co-expression network follows a scale-free topology. By analyzing the correlation between module eigengenes and sample phenotypes (e.g., pollutant concentration, exposure duration, disease status), key modules most relevant to specific biological processes can be rapidly identified. This method significantly reduces the complexity of data analysis and reveals synergistic interactions among genes at the system level. WGCNA has been widely applied to identify core gene modules and hub genes across various diseases and biological processes [9,10]. Notably, the strategy of integrating WGCNA with machine learning has become a current research hotspot [11].

Machine learning (ML), with its pattern recognition and predictive capabilities, is now widely used in biomedicine, particularly for high-dimensional feature selection and disease prediction modeling [12]. Common algorithms include Random Forest (RF), Least Absolute Shrinkage and Selection Operator (LASSO) regression, and Support Vector Machine (SVM) [13]. RF builds multiple decision trees for ensemble learning, effectively handling nonlinear relationships [14]. LASSO regression applies L1 regularization to shrink irrelevant variables to zero, enabling simultaneous selection and simplification. The SVM-based Recursive Feature Elimination (SVM-RFE) algorithm iteratively removes the least contributing features to select an optimal gene subset. Integrating ML algorithms with network toxicology, Weighted Gene Co-expression Network Analysis (WGCNA) and deep functional exploration based on Gene Set Enrichment Analysis (GSEA) provides an effective solution for mining key molecular mechanisms underlying the toxic effects of environmental pollutants from large-scale omics data [15]. However, this strategy has not yet been applied to GC research from the perspective of network toxicology of food contaminants.

Therefore, we designed a comprehensive research strategy integrating network toxicology, WGCNA, three ML algorithms (RF, LASSO, SVM-RFE), logistic regression modeling, prognostic analysis, single-cell transcriptomics, functional enrichment analysis, molecular docking, molecular dynamics simulations, and drug enrichment analysis to systematically identify core targets and potential therapeutic strategies for BaP-associated GC, followed by in vitro validation using conventional GC cell lines to identify universally significant therapeutic targets and candidate drugs for GC treatment.

First, we comprehensively collected and predicted potential molecular targets of BaP in humans using the Comparative Toxicogenomics Database (CTD). We then identified differentially expressed genes (DEGs) associated with BaP exposure by leveraging gastric tissue transcriptomic data from public databases.

Subsequently, WGCNA was applied to construct co-expression networks, screening gene modules and hub genes closely correlated with BaP-related phenotypes. Integrating DEGs with WGCNA-derived module genes, three ML algorithms were employed for feature selection and cross-validation, ultimately determining core feature genes that played decisive roles in BaP-related gastric carcinogenesis. These genes were then used to build a GC risk prediction model and assess their clinical prognostic significance. Their cell type-specific expression within the tumor microenvironment was further validated by single-cell transcriptomics. Functional enrichment analyses, including Gene Set Enrichment Analysis (GSEA) and Gene Set Variation Analysis (GSVA), were employed to uncover underlying biological pathways.

Furthermore, molecular docking and dynamics simulations were conducted to examine BaP interactions with DNA methyltransferases. Then, drug enrichment analysis was applied to identify prospective therapeutic agents. Finally, by evaluating the therapeutic potential of the enriched drugs against GC cells and its modulatory effects on key driver genes within the BaP-associated network, this study provides novel molecular targets and repurposed drugs with potential clinical value for the treatment of GC.

## 2. Results

### 2.1. Batch Processing of Datasets and Results of Differential Genes

**Figure 1:**
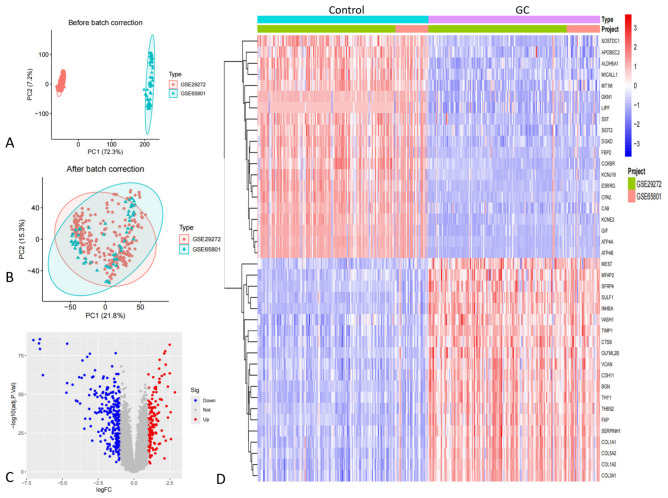
Results of batch effect processing of datasets and differential genes analysis: (**A**,**B**) Principal component analysis (PCA) plots before (**A**) and after (**B**) batch correction using the ComBat algorithm. The percentage of variance explained by PC1 and PC2 is shown in the axis labels (calculated from the data). (**C**) Volcano plots of DEG distributions contained within the merged dataset of GSE29272 and GSE65801. Red: upregulated genes; blue: downregulated genes; gray: non-significant genes; (**D**) Heatmap displaying the top 20 upregulated and downregulated DEGs.

As illustrated in Figure 1A,B, batch effects between the two GEO datasets were successfully corrected using the ComBat algorithm (see Section 4.1), making the data suitable for subsequent analyses. The percentage of variance explained by PC1 and PC2 is indicated in the axis labels of Figure 1A,B. Differential expression analysis identified 410 differentially expressed genes (DEGs) between normal and GC (GC) tissues, comprising 156 upregulated and 254 downregulated genes.

The distribution of these DEGs was visualized in the volcano plot (Figure 1C), where upregulated genes are marked in red, downregulated genes in green, and non-significant genes in gray. Furthermore, a clustered heatmap (Figure 1D) displayed the expression patterns of the top 20 upregulated and top 20 downregulated DEGs (ranked by absolute log fold change, among the total 156 upregulated and 254 downregulated DEGs) across samples, with red indicating relative upregulated genes and blue indicating downregulated genes.

### 2.2. Screening of WGCNA Module Core Genes

**Figure 2:**
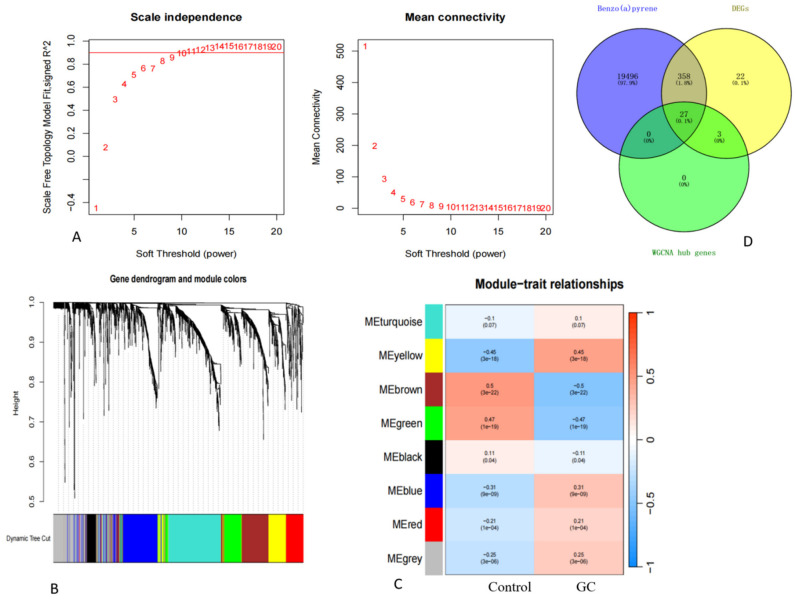
WGCNA analysis and screening of module core genes of GC. (**A**) The left illustration shows the correlation coefficient linked to various power levels, while the right illustration represents the average connection degree within the network created from these different power values. (**B**) The upper section of the figure displays the gene cluster tree derived from the dissTOM matrix established using weighted correlation coefficients. In contrast, the lower section details the distribution of genes across each module, with identical colors indicating the same module. (**C**) The vertical axis indicates the different modules, while the horizontal axis denotes each trait. The results illustrate the correlation between modules and traits, using red to signify positive correlations and green for negative correlations. (**D**) Intersection plot of known BaP-related genes, GC DEGs, and WGCNA module core genes. Purple represents BaP-related genes, green represents GC DEGs, and yellow represents WGCNA module core genes. The overlapping region (*n* = 27 genes) indicates the candidates for subsequent machine learning analysis.

We performed a WGCNA to identify gene modules associated with the clinical traits. We began by determining the soft-thresholding power to approximate a scale-free topology. Analysis of powers from 1 to 20 indicated that a value of nine optimized the scale-free model fit and mean connectivity (Figure 2A). We then constructed a hierarchical clustering tree of genes and defined co-expression modules using the dynamic tree cut method, with each module being represented by a unique color (Figure 2B). We computed module–trait relationships by correlating module eigengenes with traits of interest. This analysis identified the yellow, green, and brown modules as the most significantly associated (Figure 2C). The genes from these key modules were merged, yielding 30 non-redundant genes for further investigation.

### 2.3. Screening Important Intersected Genes of BaP and GC

According to the CTD database, 19,881 BaP-related genes were collected, which were collected from reported studies. Then, 27 intersected genes were obtained by taking the intersection of related genes of BaP, DEGs of GC, and WGCNA module core genes of GC (Figure 2D).

### 2.4. Enrichment Analysis of Important Genes of BaP on GC

**Figure 3:**
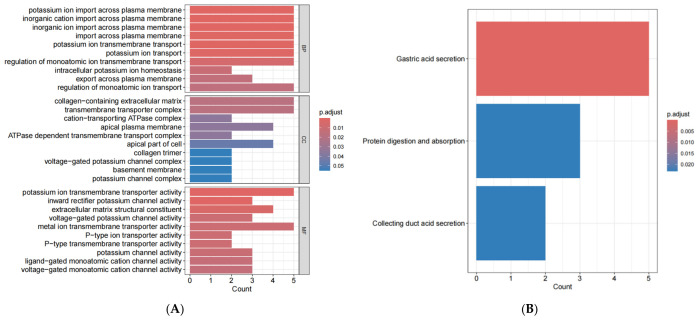
GO and KEGG enrichment analysis result of important genes of BaP-related GC (**A**). The top 10 significantly enriched GO terms (**B**). The significantly enriched KEGG pathways. The color gradient from red to blue represented the adjusted *p*-value (*p*.adjust).

The GO enrichment analysis showed that BaP-related GC was related to biological processes such as potassium ion import across the plasma membrane, inorganic cation and inorganic ion import across the plasma membrane, and potassium ion transport (see Figure 3A). The results of a KEGG enrichment analysis were shown in Figure 3B, including gastric acid secretion, protein digestion and absorption, and collecting duct acid secretion. All enriched terms were selected based on adjusted *p* < 0.05 (Benjamini–Hochberg FDR correction).

### 2.5. Screening of Hub Genes by Machine Learning

**Figure 4:**
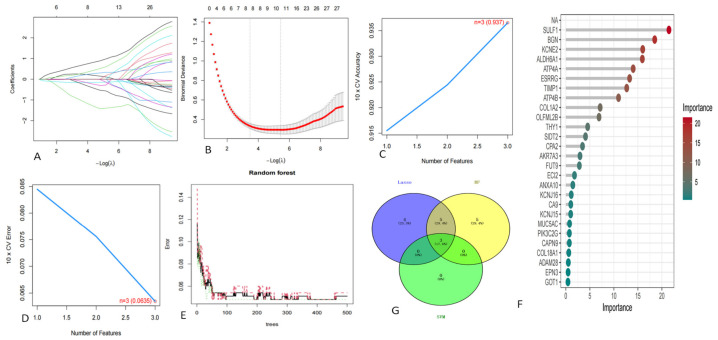
Screen hub genes of BaP-related GC by three machine learning algorithms. (**A**,**B**) LASSO regression: coefficient paths (**A**) and 10-fold cross-validation curve (**B**); (**C**) SVM-RFE: cross-validated accuracy vs. number of features; optimal subset (3 genes) marked in red; (**D**) 10-fold cross-validation error of SVM-RFE; (**E**) the correlation between the total number of trees in the random forest and the error rates; (**F**) Random Forest variable importance. Genes were ranked by mean decrease Gini (MDG); (**G**) Venn diagram showing the intersection of genes selected by the three algorithms. Green: SVM-RFE (3 genes); blue: LASSO (12 genes); red: Random Forest (13 genes). The overlapping region (3 genes: KCNE2, SULF1, TIMP1) represent the final hub genes.

To identify core feature genes playing decisive roles in BaP-related GC, three machine learning algorithms, including LASSO, SVM-RFE, and Random Forest (RF), were applied for feature selection and cross-validation using the 27 pre-filtered candidate genes (intersection of CTD, DEGs, and WGCNA genes). In the LASSO regression analysis, the optimal regularization parameter (λ) was determined via 10-fold cross-validation, which selected 12 genes as the optimal set (Figure 4A,B). This configuration balanced model complexity and predictive performance, achieving the lowest mean cross-validation error and ensuring effective regularization. The SVM-RFE analysis indicated that retaining three genes resulted in the smallest error rate, reflecting optimal predictive performance (Figure 4C,D). For the Random Forest model, the optimal number of trees was set between 100 and 450, corresponding to the minimum cross-validation error (Figure 4F). Feature importance was evaluated using the mean decrease Gini (MDG) metric, which quantified the average reduction in node impurity contributed by each gene across all decision trees. Based on the empirical distribution of MDG scores, we applied a threshold of MDG > 3, which captured a natural elbow in the sorted importance values and retained 13 candidate genes (Figure 4E,F). Finally, the intersection (rather than the union) of the genes selected by the three machine learning algorithms was used to identify three hub genes (Figure 4G), as this consensus approach maximally reduces false positives and ensures robustness across distinct algorithmic mechanisms. The intersection genes revealed three hub genes (KCNE2, SULF1, and TIMP1) as key mediators in BaP-related gastric carcinogenesis.

### 2.6. Construction of the Risk Prediction Model

A binary logistic regression model was constructed using the three hub genes (KCNE2, SULF1, and TIMP1) to assess GC risk. The risk score was calculated as follows:

Risk Score = −0.664 × KCNE2 + 1.078 × SULF1 + 1.677 × TIMP1 − 22.652

**Figure 5:**
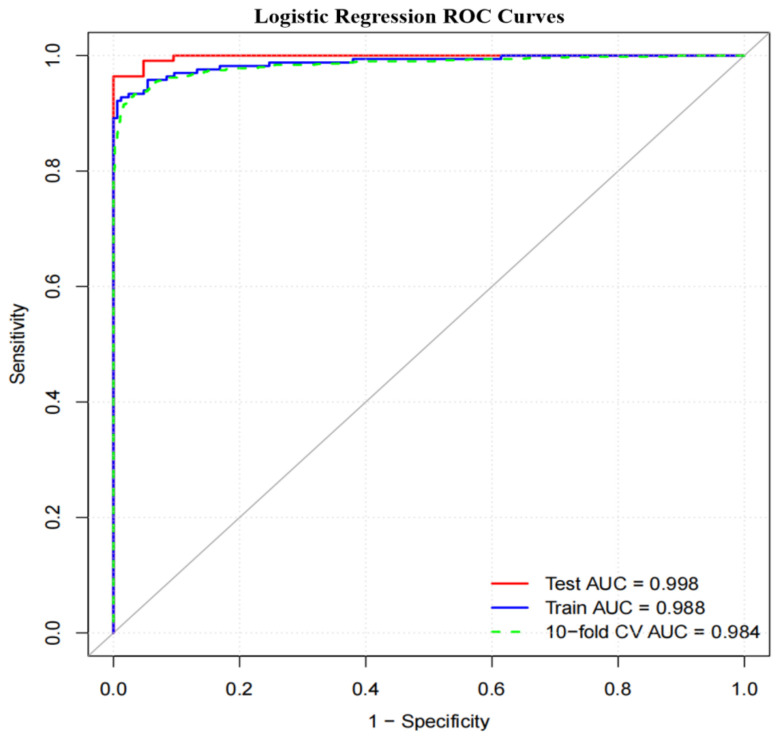
Performance evaluation of the GC risk model. The model was trained on the merged GSE29272 and GSE65801 dataset (*n* = 332) and validated externally on GSE54129 (*n* = 132). Blue curve: training set performance; red curve: external test set; green dashed curve: 10-fold cross-validation performance of training set.

Table 1 lists the results of logistic regression analysis of hub genes for GC risk prediction. The elevated expression of KCNE2 served as a protective factor (OR = 0.515, 95% CI: 0.374–0.682), while upregulation of SULF1 and TIMP1 were identified as risk factors, with ORs of 2.940 (95% CI: 1.439–6.461) and 5.351 (95% CI: 2.020–16.743), respectively. To evaluate the stability of the OR estimates, we performed bootstrap validation with 1000 resamples. The bias-corrected accelerated (BCa) confidence intervals were consistent with the original intervals (e.g., TIMP1: bootstrap mean OR = 7.313, 95% BCa CI = 1.975–15.820), confirming the statistical robustness of the associations. The wide CI for TIMP1 likely reflects the limited sample size or inherent biological variability. No significant multicollinearity was detected among the three predictors (VIF values: KCNE2 = 1.016, SULF1 = 1.329, TIMP1 = 1.316). Additionally, as shown in Figure 5, the model demonstrated outstanding discriminative ability, achieving an AUC of 0.988 in the training set (mean 10-fold cross-validated AUC = 0.984) and an AUC of 0.998 in the external test set (GSE54129), indicating robust generalizability. These results suggested that KCNE2, SULF1, and TIMP1 not only constituted a high-performance predictive signature for GC risk but also played critical biological roles in GC pathogenesis, as supported by their significant ORs.

### 2.7. Clinical Relevance and Prognostic Analysis of Hub Genes of BaP-Related GC

**Figure 6:**
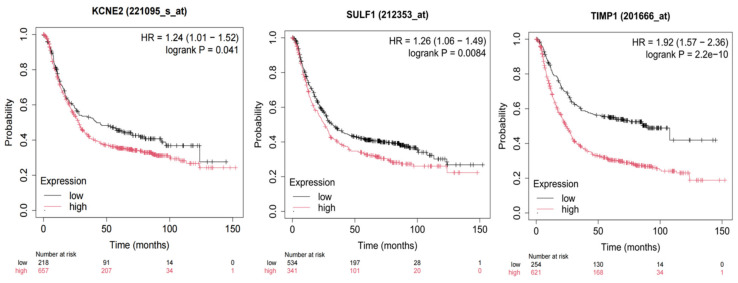
Prognostic value of hub genes of BaP in GC.

To evaluate the clinical relevance of the identified hub genes, survival analysis was performed to assess the association between their expression levels and overall survival in GC patients (Figure 6). Kaplan–Meier survival curves demonstrated that patients with high expression of each hub gene (red lines) exhibited significantly shorter overall survival compared with those with low expression (black lines). The hazard ratios (HRs) for high-expression groups were 1.24 (95% CI: 1.01–1.52, *p* = 0.041) for KCNE2, 1.26 (95% CI: 1.06–1.49, *p* = 0.008) for SULF1, and 1.92 (95% CI: 1.57–2.36, *p* = 2.2 × 10^−10^) for TIMP1. These results consistently indicated that elevated expression of SULF1 and TIMP1 was associated with unfavorable prognosis in GC, with TIMP1 showing the strongest association with poor clinical outcomes. For KCNE2, although its expression was downregulated in GC tissues, higher expression within tumors paradoxically correlated with worse prognosis, suggesting that KCNE2 may switch from a tumor-suppressive role in incidence to a pro-aggressive function during tumor progression. Such context-dependent dual roles are well recognized in cancer biology.

### 2.8. Single-Cell Expression Profiles of Hub Genes of BaP-Related GC

**Figure 7:**
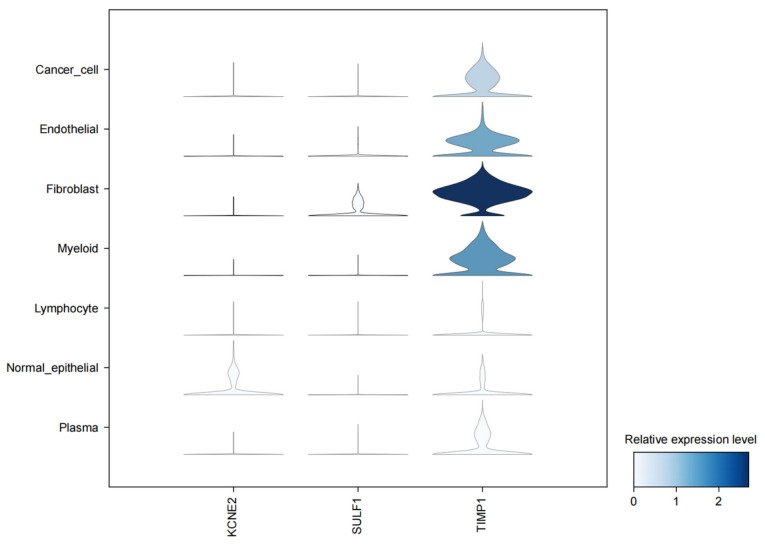
Single-cell expression patterns of 3 hub genes in GC tissues based on GSE210347 dataset. Violin plots showing the expression distribution of these genes across a variety of cellular populations. The horizontal axis and the color gradient represented the relative expression level (scale bar on the right). The width of the violin reflected the density of cells at a given expression level.

Cell type-specific deconvolution of the tumor microenvironment (GSE210347) delineated unique expression profiles for the identified hub genes (Figure 7). In Figure 7, violin plots were used to display the distribution of relative expression levels across cell types. The y-axis (and the color gradient, when applicable) represented the relative expression level. The risk gene TIMP1 exhibited a br oad expression pattern, showing significant upregulation in fibroblasts, myeloid cells, and cancer cells and endothelial cells. SULF1 expression was highly specific to and enriched in fibroblasts. Conversely, the protective factor KCNE2 was exclusively abundant in normal epithelial cells and was largely absent in all other cellular components, most notably in the cancer cells.

### 2.9. GSVA for Hub Genes of BaP-Related GC

To elucidate the functional pathways associated with the identified hub genes, Gene Set Variation Analysis (GSVA) was performed using GO and KEGG gene sets. The top five upregulated and downregulated pathways for each hub gene are summarized in Supplementary Table S1, with detailed statistics. GSVA revealed that high KCNE2 expression was strongly associated with the suppression of cellular differentiation and developmental signaling. Key downregulated biological processes included phenotypic switching, reversible differentiation, and negative regulation of muscle cell differentiation (all *p* < 0.001). Concurrently, KCNE2 upregulation was linked to enhanced ion channel activity, specifically negative regulation of delayed rectifier potassium channel activity, vesicle docking, and vesicle docking involved in exocytosis. KEGG analysis further indicated a profound metabolic shift, with significant activation of multiple amino acid and energy metabolism pathways, such as nitrogen metabolism, histidine metabolism, and glycolysis/gluconeogenesis. In contrast, several cancer-related pathways, including bladder cancer and basal cell carcinoma, as well as signaling pathways like the notch signaling pathway, were significantly inhibited. The enrichment profile for SULF1 highlighted its central role in extracellular matrix (ECM) organization and developmental reprogramming. The most significantly activated pathways included GOBP negative regulation of extracellular matrix disassembly, GOMF collagen binding, and KEGG ECM receptor interaction. Developmental processes such as endodermal cell differentiation and the TGF beta signaling pathway were also activated. Notably, KEGG pathways in cancer were among the top upregulated pathways. Conversely, high SULF1 expression was characterized by a coordinated suppression of nucleic acid metabolism and repair mechanisms, including KEGG DNA replication, base excision repair, and GOBP mRNA modification. Amino acid catabolic pathways, particularly valine, leucine, and isoleucine degradation, were also significantly downregulated. High TIMP1 expression exhibited a dual pattern of strong pathway activation and suppression. It was prominently associated with the upregulation of pathways involved in ECM remodeling, oncogenic signaling, and immune response. The most significantly enriched terms included KEGG complement and coagulation cascades, ECM receptor interaction, glycosaminoglycan biosynthesis chondroitin sulfate, and TGF beta signaling pathway. KEGG pathways in cancer and cytokine–cytokine receptor interactions were also activated. In contrast, a broad and pronounced downregulation of core metabolic processes was observed. This included severe suppression of amino acid catabolism (valine, leucine, and isoleucine degradation, central carbon metabolism (citrate cycle TCA cycle; pyruvate metabolism), and fatty acid metabolism (peroxisome), a finding corroborated by GO analysis.

### 2.10. Molecular Docking and Molecular Dynamics Results of BaP and DNMTs

**Figure 8:**
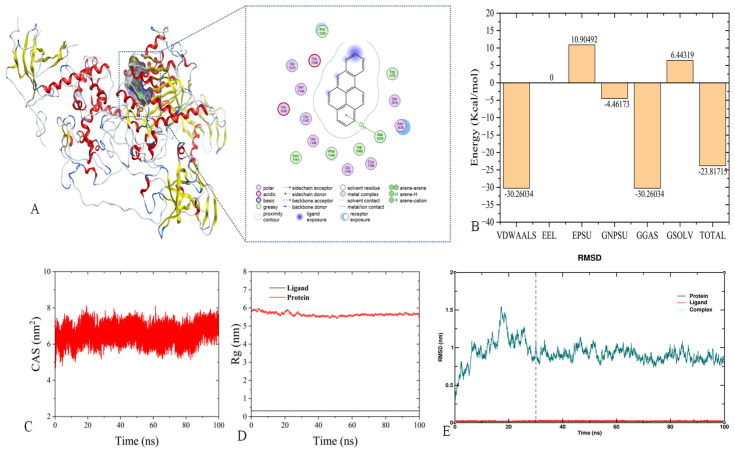
The analysis results of molecular docking and molecular dynamics simulation of BaP-DNMT1. (**A**) The 3D and 2D docking modes; (**B**) binding free energy in the dynamic process; (**C**) variation in the contact area surface between BaP and DNMT1 in the dynamic process; (**D**) variation in the gyration radius of BaP and DNMT1 in the dynamic process; (**E**) root-mean-square deviation (RMSD) changes for protein, ligand, and the complex of protein–ligand during a 100 ns molecular dynamics simulation. The vertical dashed line at 30 ns indicated the start of the equilibrium phase.

**Figure 9:**
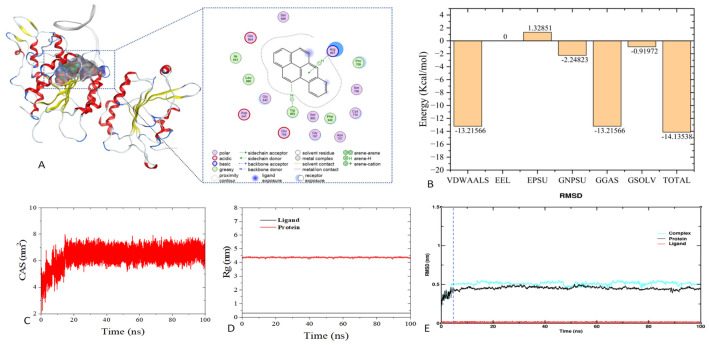
The analysis results of molecular docking and molecular dynamics simulation of BaP-DNMT3A. (**A**) The 3D and 2D docking modes; (**B**) binding free energy in the dynamic process; (**C**) variation in the contact area surface between BaP and DNMT3A in the dynamic process; (**D**) variation in the gyration radius of BaP and DNMT3A in the dynamic process; (**E**) root-mean-square deviation (RMSD) changes for protein, ligand, and the complex of protein–ligand during a 100 ns molecular dynamics simulation. The vertical dashed line at 5 ns indicated the start of the equilibrium phase.

GSVA enrichment analysis revealed that the three genes KCNE2, SULF1, and TIMP1 were significantly involved in processes such as epigenetic regulation, extracellular matrix reorganization, TGF-β signaling activation, and metabolic reprogramming, with associations with DNA methylation regulation. Consistent with existing reports, BaP can induce upregulation of DNA methyltransferases DNMT1 and DNMT3A, which may catalyze methylation state alterations in the promoter regions of multiple genes, including KCNE2, SULF1, and TIMP1, ultimately mediating transcriptional regulation and signaling pathway reprogramming. To explore whether BaP can interact with these methyltransferases, we performed molecular docking and molecular dynamics simulations. As shown in Figure 8 and Figure 9, the docking results demonstrated that BaP stably bound within the active catalytic pocket of both DNMT1 and DNMT3A, with predicted binding free energies of −6.249 kcal/mol and −6.111 kcal/mol, respectively.

Subsequent MD simulations further revealed the dynamic stability and conformational behavior of the BaP-DNMT complexes. The stability of the BaP-DNMT complexes was assessed through molecular dynamics simulations. For both DNMT1 and DNMT3A, the protein–ligand contact surface area remained stable throughout the simulation trajectory (Figure 8C and Figure 9C), indicating sustained and close interaction at the binding pocket. This was further supported by the radius of gyration (Rg), which stabilized immediately after simulation initiation (Figure 8D and Figure 9D), suggesting no significant unfolding or global change in the tertiary structure of the protein upon ligand binding. Root-mean-square deviation (RMSD) analysis confirmed the rapid convergence and stability of the complexes. The RMSD of the DNMT1-BaP complex plateaued within 25–30 ns and remained stable thereafter; the equilibrium portion for analysis was from 30 ns to 100 ns in Figure 8E. For DNMT3A-BaP, the RMSD stabilized within a few nanoseconds, and the equilibrium portion was from 5 ns to 100 ns in Figure 9E. In both cases, the RMSD trajectory of the complex closely mirrored that of the protein alone, and the ligand RMSD showed minimal fluctuation, collectively demonstrating a stable binding pose with a rigid ligand conformation. Root-mean-square fluctuation (RMSF) analysis was performed to evaluate residue-level flexibility. For DNMT1, chain A (containing the BaP binding pocket) exhibited generally lower RMSF values compared with chain B, indicating that BaP binding induces local structural stabilization of the binding pocket. The active site residues in chain A displayed reduced flexibility relative to other regions, suggesting a rigidified conformation upon ligand binding (Supplementary Figure S1). For DNMT3A, four chains showed distinct dynamic patterns. The BaP binding site was located in chain C. Chain A displayed the highest conformational flexibility (mean RMSF ≈ 3.5 Å), while its homolog chain D was the most rigid. Chain C (ligand-bound) showed higher flexibility than its counterpart chain B, consistent with the presence of more active residues interacting with BaP (Supplementary Figure S2). Figure S3 showed the temporal evolution of intraprotein hydrogen bonds within DNMT1 and DNMT3A1 during the 100 ns MD simulations. As the simulations progressed, the number of internal hydrogen bonds rapidly increased and plateaued into stable fluctuations after approximately 20–40 ns, indicating that both proteins gradually adopted compact conformations and reached structural equilibrium, with well-preserved secondary structures and strong internal stability. To further clarify the ligand binding mechanism, we quantified the hydrogen bonds formed between BaP and DNMT1/DNMT3A1 over the trajectory. The results showed that BaP formed virtually no stable hydrogen bonds with either protein (average number ≈ 0) throughout the 100 ns simulation. This observation is fully consistent with the intrinsic physicochemical properties of BaP: as a typical polycyclic aromatic hydrocarbon, the molecule lacks strongly electronegative atoms (such as O and N) and therefore does not possess the capacity to act as a hydrogen bond donor or acceptor. Consequently, the binding driving forces primarily rely on nonpolar interactions. Our analysis concludes that the binding of BaP to DNMT1 and DNMT3A1 is likely dominated by hydrophobic interactions, van der Waals forces, and potential π–π stacking, rather than hydrogen bonding. The binding free energy calculated by the MM-PBSA method further confirmed the spontaneous binding process, with values of −23.82 kcal/mol for DNMT1 and −14.14 kcal/mol for DNMT3A (Figure 8B and Figure 9B).

These structural insights suggest that BaP may act as a competitive inhibitor, potentially interfering with the binding of DNMT substrate. This provides a mechanistic basis for the observed methylation changes in KCNE2, TIMP1, and SULF1, thereby linking BaP exposure to epigenetic dysregulation in gastric carcinogenesis.

### 2.11. Drug Enrichment Analysis of BaP-Related GC

**Figure 10:**
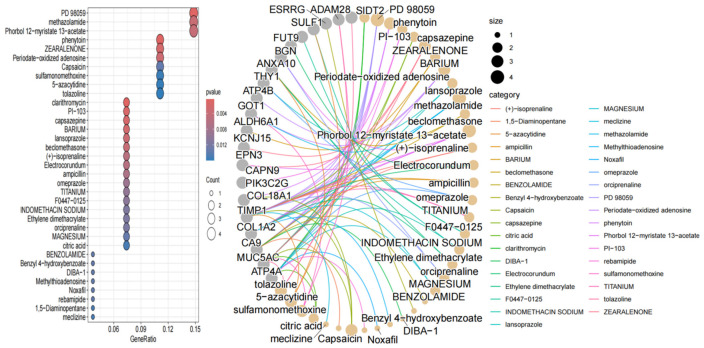
Drug enrichment analysis results of BaP-related GC.

Drug enrichment analysis was performed on the intersected genes of BaP and GC. As illustrated in Figure 10, several compounds, including PD-98059, methazolamide, phorbol 12-myristate 13-acetate, and 5-azacytidine, were significantly enriched among these genes, indicating their potential as core therapeutic agents for GC. Notably, 5-azacytidine was identified as a drug molecule targeting both TIMP1 and SULF1, highlighting its particular relevance to the BaP-related GC gene signature.

### 2.12. Cell Validation of 5-Azacytidine Against GC

**Figure 11:**
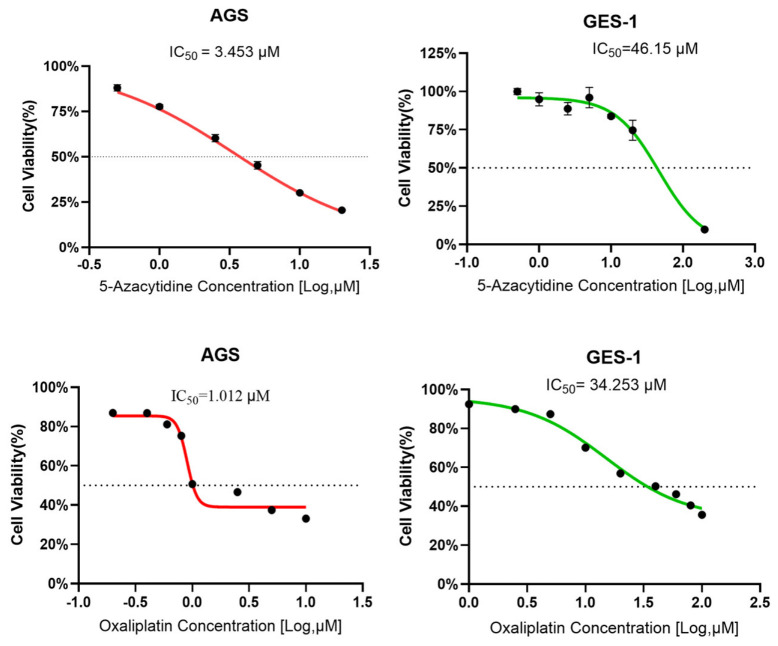
Dose-dependent inhibition of AGS and GES-1 cell viability by 5-azacytidine and oxaliplatin, with calculated IC_50_ values and selectivity indices (SIs).

As shown in Figure 11, using the clinical chemotherapeutic agent oxaliplatin as a positive control, both 5-azacytidine and oxaliplatin dose-dependently inhibited the viability of AGS GC cells and GES-1 normal gastric epithelial cells. The half-maximal inhibitory concentration (IC_50_) of oxaliplatin was 1.012 μM for AGS cells and 34.253 μM for GES-1 cells, yielding a selectivity index (SI) of 33.85, indicating favorable tumor selectivity consistent with its established antitumor properties. For 5-azacytidine, the IC_50_ was 3.453 μM for AGS cells and 46.15 μM for GES-1 cells, with an SI of 13.37. Both compounds exhibited SI values exceeding 10, demonstrating favorable tumor selectivity over normal gastric epithelial cells.

**Figure 12:**
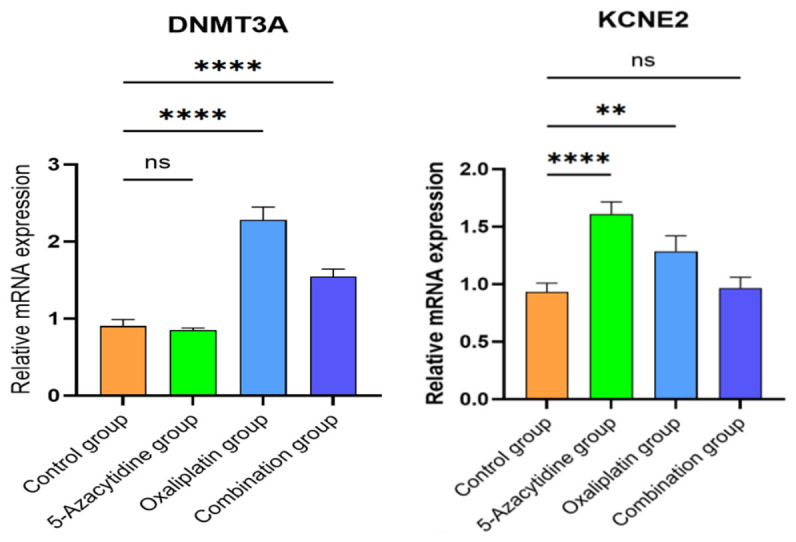
Effects of 5-azacytidine, oxaliplatin, and their combination on the expression of DNMT3A and KCNE2 in AGS cells as determined by RT-qPCR. Control group: untreated AGS cells; 5-azacytidine group: AGS cells treated with 5-azacytidine at IC_30_; oxaliplatin group: AGS cells treated with oxaliplatin at IC_30_; combination group: AGS cells treated with oxaliplatin at IC_20_ combined with 5-azacytidine at IC_20_. Data are presented as the mean ± SD from three independent biological replicates (*n* = 3), each with three technical replicates. Statistical analysis was performed using a one-way ANOVA. ^ns^ *p* > 0.05, ** *p* < 0.01, **** *p* < 0.0001 compared with the control group.

To compare the regulatory effects of single agent versus combined treatment under comparable levels of cytotoxicity, AGS cells were treated with single agents at their respective IC_30_ concentrations (5-azacytidine and oxaliplatin) or with the combination at the IC_20_ concentration of each agent. RT-qPCR results (Figure 12) revealed that, compared with the control group, 5-azacytidine alone (IC_30_) downregulated DNMT3A mRNA expression while significantly upregulating KCNE2 expression (*p *< 0.05). Oxaliplatin alone (IC_30_) also significantly upregulated KCNE2 expression (*p *< 0.05) but concomitantly led to a marked increase in DNMT3A expression (*p *< 0.05). In the combination group (each at IC_20_), expression levels of both DNMT3A and KCNE2 returned to near those of the control group.

Protein validation (Figure S4) confirmed that 5-azacytidine, oxaliplatin, and their combination all significantly reduced DNMT3A protein (*p* < 0.001), with the combination showing the strongest effect. For KCNE2, oxaliplatin significantly elevated its protein (*p* < 0.05), while 5-azacytidine had no significant protein effect despite mRNA upregulation, indicating possible post-transcriptional regulation. The combination decreased KCNE2 protein level (*p* < 0.05). Therefore, 5-azacytidine may effectively contributed to the DNMT3A–KCNE2 axis at both transcriptional and protein levels for DNMT3A, and oxaliplatin independently supported KCNE2 as a protective factor. Collectively, these results suggest that KCNE2 may represent a potential novel protective target and that 5-azacytidine could be a repurposed candidate for gastric cancer, consistent with our computational discovery.

## 3. Discussion

Gastric cancer (GC) remains a common malignancy worldwide. BaP, a foodborne carcinogen from high-temperature processing, directly targets the stomach via dietary exposure. By integrating network toxicology, multi-omics, machine learning (LASSO, SVM-RFE, Random Forest), and experimental validation, we systematically identified therapeutic targets and repurposed drugs for GC from the perspective of BaP-induced molecular networks. Machine learning algorithms identified KCNE2, SULF1, and TIMP1 as hub genes linking BaP exposure to GC. A risk prediction model based on these three genes achieved AUCs of 0.988 (training) and 0.998 (external validation), demonstrating strong generalizability and clinical potential. Moreover, these genes exhibited significant biological relevance in GC pathogenesis, supported by substantial odds ratios.

Notably, KCNE2 exhibited a significant protective effect against GC (OR = 0.515, 95% CI: 0.383–0.692). This OR suggests that each one-unit increase in KCNE2 expression decreases the risk of developing GC by approximately 48.5%. KCNE2 encodes a β-subunit of potassium ion channels and is highly expressed in gastric parietal cells, where it participates in regulating gastric acid secretion [16]. Consistent with our findings, multiple previous studies have confirmed significant downregulation or loss of KCNE2 expression in GC tissues [17,18]. Mouse knockout models of KCNE2 developed precancerous lesions such as gastric mucosal hyperplasia and gastritis, ultimately progressing to GC [19]. Taken together, these findings indicate that low KCNE2 expression or loss of function is a critical risk factor for GC development.

Mechanistically, previous studies have shown that BaP induces hypermethylation of the KCNE2 promoter region, leading to its transcriptional silencing [20], which is consistent with the downregulation of KCNE2 observed in GC samples in our study. GSVA further revealed that samples with low KCNE2 expression exhibited downregulation of notch signaling, axon guidance, and multiple tumor-associated pathways, whereas amino acid and fatty acid metabolism pathways were activated. These findings suggest that KCNE2 deficiency may promote GC progression by disrupting developmental signals and reshaping the tumor metabolic microenvironment.

In contrast, SULF1 (OR = 2.940, 95% CI: 1.399–6.179) and TIMP1 (OR = 5.351, 95% CI: 1.879–15.238) were identified as significant risk factors for GC. The odds ratio for SULF1 indicates that each one-unit increase in its expression corresponds to an approximately 194.0% increase in GC risk, underscoring its critical role in tumorigenesis. SULF1 is an extracellular sulfatase that specifically removes 6-O-sulfate groups from heparan sulfate proteoglycans (HSPGs), thereby modulating multiple growth factor signaling pathways, including Wnt, FGF, VEGF, and HGF [21]. Studies on GC patients have demonstrated a marked upregulation of SULF1 expression, which was directly associated with poor prognosis [22], which is consistent with our findings that elevated SULF1 levels significantly reduced patient survival. Previous research suggested that BaP may induce long-term oncogenic activity by modulating the epigenetic status of SULF1. This aligned with the high SULF1 expression phenotype observed in our study [23].

Our GSVA analysis further supported the oncogenic role of SULF1. Samples with high SULF1 expression exhibited significant activation of extracellular matrix (ECM)-related pathways, including ECM–receptor interaction and collagen binding, as well as developmental and differentiation pathways such as endoderm cell differentiation and the TGF-β signaling pathway. It was also reported that SULF1 promoted GC metastasis through the TGF-β signaling pathway [22]. Given that ECM remodeling is a critical step in tumor invasion and metastasis, and the fact that TGF-β signaling was a pro-tumorigenic factor in advanced GC, these functional insights collectively established SULF1 as a key oncogenic driver in BaP-related GC progression.

TIMP1 exhibited the strongest risk effect among the three genes (OR = 5.351), with each one-unit increase in its expression associated with an approximately 435.1% increase in GC risk. Traditionally considered a tumor suppressor by inhibiting MMPs to block ECM degradation, recent studies have revealed its pro-tumorigenic functions, including promoting proliferation, inhibiting apoptosis, inducing angiogenesis, and remodeling the immune microenvironment [24]. In GC cohorts, TIMP1 was highly expressed in both tissue and serum and correlated with reduced overall survival, consistent with our prognostic findings and validating its utility as a prognostic biomarker [25,26].

Notably, previous studies have shown that BaP upregulates TIMP1 expression by modulating its methylation status, consistent with the high TIMP1 expression observed in our study [27]. Mechanistically, BaP affects DNA methylation in the TIMP1 promoter region, enhancing its transcriptional activity, which may be a key event driving sustained TIMP1 activation in BaP-related gastric carcinogenesis.

Our GSVA analysis confirmed the oncogenic role of TIMP1. In samples with high TIMP1 expression, ECM-related pathways (e.g., ECM–receptor interaction, focal adhesion) and tumor-associated pathways (e.g., TGF-β, mTOR) were significantly activated, and immune microenvironment pathways (e.g., cytokine–cytokine receptor interaction, complement cascade) were also enriched. Meanwhile, multiple amino acid metabolism pathways (e.g., valine, leucine, and isoleucine degradation) and core energy metabolism pathways (e.g., TCA cycle, pyruvate metabolism) were consistently downregulated.

The broad expression of the risk gene TIMP1 in fibroblasts and myeloid and cancer cells positioned it as a key modulator of stromal and immune interactions. The specific enrichment of SULF1 in cancer-associated fibroblasts pointed to a stromal-driven mechanism for its pro-tumorigenic effects. Conversely, the exclusive expression of the protective factor KCNE2 in normal epithelium and its universal loss in tumor cells provide a cellular basis for its role as a guardian whose silencing was critical for carcinogenesis. These patterns validated our multi-omics findings and clarify the cell populations driving the hub gene-mediated pathogenesis.

GSVA enrichment analysis indicated that the aberrant expression of the three hub genes (KCNE2, SULF1, and TIMP1), which is involved in key cancer-related processes, was potentially linked to DNA methylation changes. This observation prompted us to investigate whether BaP induces epigenetic dysregulation in gastric carcinogenesis by targeting DNA methyltransferases. This was strongly supported by existing literature indicating that BaP can upregulate DNMT1 and DNMT3A, enzymes responsible for catalyzing promoter methylation [28].

To verify the mechanism between BaP exposure and its epigenetic consequences, we employed computational structural biology approaches. Molecular docking simulations confirmed that BaP can stably occupy the active pocket of both DNMT1 and DNMT3A with moderate yet specific binding affinities, which was consistent with the chronic nature of epigenetic effects. The dynamic behavior of these complexes, as revealed by molecular dynamics simulations, further solidified this finding. The rapid equilibration of the RMSD and the highly favorable binding free energies calculated by MM-PBSA unequivocally indicated that the binding was spontaneous and stable.

Therefore, these structural insights indicated that BaP may function as a competitive inhibitor by binding to DNMTs. This interaction might interfere with their catalytic activity or specificity, potentially contributing to the aberrant transcription of KCNE2, SULF1, and TIMP1. Thus, our study offered potential new targets for intervening in the carcinogenic process. While the causality between BaP and the three genes needed to be verified in exposure-matched samples, this was a question beyond the discovery-oriented scope of the present work.

Furthermore, drug enrichment analysis identified the DNMT inhibitor 5-azacytidine as a potential therapeutic agent targeting TIMP1 and SULF1. As the first FDA-approved epigenetic drug originally used for myelodysplastic syndromes and other hematological malignancies, 5-azacytidine exerts its antitumor effects by inhibiting DNA methyltransferases and reversing CpG island hypermethylation in tumor suppressor gene promoter regions, thereby restoring their expression [29,30]. Our findings were consistent with recent research suggesting that combining the DNMT inhibitor 5-azacytidine with MEK/ERK inhibitors represented a promising strategy for GC patients [31]. This provided compelling pharmacological evidence supporting the pivotal role of DNA methylation regulation in BaP-related gastric carcinogenesis. However, the relationship between 5-azacytidine and the protective factor KCNE2, a tumor suppressor that can be therapeutically restored, still remains unexplained.

Finally, our in vitro results demonstrated that 5-azacytidine exhibited favorable selectivity for AGS GC cells (SI = 13.37). It was consistent with our drug enrichment analysis that identified DNMT inhibitors as top candidates for targeting BaP-associated GC pathways. RT-qPCR further revealed that 5-azacytidine monotherapy downregulated DNMT3A and significantly upregulated the protective gene KCNE2, validating that the DNMT3A–KCNE2 axis, which we identified as a central node in BaP-associated epigenetic disruption from multi-omics, is functionally targetable in GC cells.

At the protein level, 5-azacytidine significantly reduced DNMT3A but did not significantly increase KCNE2, despite clear mRNA upregulation. This discordance likely reflects post-transcriptional regulation (e.g., translation inefficiency or rapid protein turnover) that does not negate the protective role of KCNE2. Indeed, oxaliplatin alone significantly increased KCNE2 at both mRNA and protein levels (*p* < 0.05), independently supporting KCNE2 as a protective factor whose upregulation correlates with anti-cancer activity.

Notably, while oxaliplatin monotherapy also upregulated KCNE2 expression, it unexpectedly increased DNMT3A mRNA levels. At the protein level, however, oxaliplatin still significantly reduced DNMT3A (*p* < 0.001), albeit to a lesser extent than 5-azacytidine. This mRNA–protein reversal suggests that oxaliplatin may activate a post-translational degradation pathway (e.g., ubiquitin–proteasome) that overrides transcriptional upregulation, a known response to DNA damage. This finding may explain why low-dose combination therapy (each at IC_20_) failed to synergistically enhance KCNE2 expression. Moreover, the combination unexpectedly reduced KCNE2 protein (*p* < 0.05) despite unchanged mRNA, indicating that low-dose co-treatment may trigger alternative degradation mechanisms. The concurrent “normalization” of DNMT3A and KCNE2 in the combination group suggests antagonistic rather than synergistic interactions at this dose.

Therefore, our in vitro experimental results suggest that KCNE2 may represent a potential novel protective target and that 5-azacytidine could be a repurposed candidate for gastric cancer, consistent with our computational discovery.

## 4. Materials and Methods

### 4.1. Data Processing and Differentially Expressed Genes (DEGs) Analysis

Gene expression datasets related to GC were retrieved from the Gene Expression Omnibus (GEO) database (https://www.ncbi.nlm.nih.gov/geo/, accessed on 10 February 2025) using the keywords “GC” and “Normal” with the organism restricted to *Homo sapiens*. The inclusion criteria for dataset selection were: (1) datasets generated from microarray expression profiling; (2) human tissue samples; (3) sample size greater than 10 per group; and (4) availability of both GC and matched normal control samples. Based on these criteria, three datasets (GSE29272, GSE65801, and GSE54129) were selected for further analysis. GSE29272 and GSE65801 were merged to form the primary cohort for identifying DEGs and core gene analysis, comprising 166 GC samples and 166 normal controls. GSE54129, including 111 GC and 21 normal samples, was reserved as an independent external validation cohort to assess the clinical diagnostic performance of candidate genes.

To ensure data integrity and comparability, rigorous preprocessing was applied. Initial evaluation of batch effects between the two merged datasets was conducted using principal component analysis (PCA). Batch effects were subsequently corrected using the ComBat algorithm implemented in the sva R package (version R4.4.0). The raw microarray data were normalized employing the Robust Multi-array Average (RMA) method, which encompasses background correction, quantile normalization, and log2 transformation, thereby enhancing data consistency and comparability [32].

Differential expression analysis between GC and normal samples was performed using the limma package, which applies linear models to accommodate complex experimental designs and covariates [33]. DEGs were identified based on thresholds of |log2 fold change (FC)| > 1 and Benjamini–Hochberg false discovery rate (FDR) adjusted *p*-value < 0.05 to ensure statistical robustness and biological relevance [34]. Volcano plots illustrating DEG distributions were generated using the ggplot2 R package. Additionally, known BaP-related genes were retrieved from the Comparative Toxicogenomics Database (CTD) [35].

### 4.2. Identification of Key Genes Related to Clinical Traits

Weighted Gene Co-expression Network Analysis (WGCNA) was performed to explore the key genes and modules related to clinical traits using the WGCNA R package. An appropriate soft-thresholding power was selected to achieve scale-free topology (scale-free fit index R^2^ ≥ 0.9). Gene modules were identified via hybrid dynamic tree-cutting based on a topological overlap matrix (TOM) with minimum module size = 60 genes. Modules exhibiting significant trait correlations (*p* < 0.05) were retained for downstream analysis [36].

### 4.3. Potential Targets of BaP-Related GC

To investigate the role of BaP in GC, a Venn diagram was constructed to visualize the overlap between known BaP-related genes and hub genes of GC. The analysis integrated three gene sets: known BaP-related genes retrieved from CTD (https://ctdbase.org/about/dataCategory.jsp, accessed on 20 February 2025), differentially expressed genes (DEGs) identified from GC transcriptomic data, and hub genes derived from WGCNA. Genes present in the common overlap of all three sets were thereby identified as high-confidence candidates mediating BaP’s effects in GC.

### 4.4. GO and KEGG Analyses

The R package clusterProfiler was used for GO and KEGG analyses. It is a powerful tool for functional enrichment analysis and gene set interpretation, supporting functional characteristics of coding and non-coding genomics data for thousands of species. Annotations were based on the GO knowledgebase [37] and KEGG pathway database [38]. Enrichment significance was performed using the hypergeometric distribution. All resulting *p*-values were adjusted for multiple comparisons using the Benjamini–Hochberg false discovery rate (FDR) method, and adjusted *p* < 0.05 was considered statistically significant. The top 10 cellular components, molecular functions, biological processes, and KEGG pathways were visualized by the enrichplot package, a versatile tool for creating plots like bubble plots and dot plots, enabling flexible data presentation.

### 4.5. Screening Hub Genes of BaP-Related GC via Machine Learning

To identify key genes implicated in BaP-related gastric carcinogenesis, we employed three independent machine learning algorithms, RF, LASSO, and SVM-RFE, strictly within the training cohort.

The LASSO model was constructed with the glmnet package (from R package, version 4.1.10), applying L1 regularization to eliminate insignificant coefficients. The optimal regularization parameter λ was determined through 10-fold cross-validation using cv.glmnet() with type.measure = “deviance”; both λ.min (minimum mean cross-validated error) and λ.1se (largest λ within one standard error) were considered. The parameter grid comprised 100 λ values automatically generated by glmnet. Stratified folds were created using caret::createFolds() to preserve case/control proportions, with the random seed set to 123 for reproducibility.

For SVM-RFE, a recursive feature elimination was performed using the caret and e1071 R packages with linear SVM (svmLinear, kernel = “linear”, scale = FALSE). Feature subsets were evaluated using 10-fold cross-validation repeated 10 times to ensure robust performance estimation. The rfeControl function was configured with method = “cv”, number = 10, and repeats = 10; the optimal feature subset was selected as the one with the minimum cross-validated error.

The RF model was built and optimized using the randomForest (version 4.7.1) and caret packages. The mtry parameter (number of variables randomly sampled at each split) was tuned via 10-fold cross-validation using caret::train() with method = “rf”. The number of trees (ntree) was set to 500 based on convergence of the out-of-bag (OOB) error. Variable importance was assessed by mean decrease Gini (MDG). Candidate genes were selected based on an empirically determined MDG threshold (>3), which corresponded to a natural elbow in the sorted importance values and ensured a balanced number of features for downstream intersection.

Finally, the hub genes were defined as the overlapping genes selected by all three algorithms. This consensus approach minimizes false positives and ensures robustness.

### 4.6. Construction and Validation of the GC Risk Prediction Model

A binary logistic regression (LR) model was implemented using the glm function in R to estimate the regression coefficients for the hub genes and construct a GC risk prediction model. The expression levels of hub genes were included as independent variables, with the sample status (GC vs. normal) serving as the binary dependent variable. Multicollinearity was assessed using the variance inflation factor (VIF) calculated via the vif function from the car R package. Odds ratios (ORs) and their corresponding 95% confidence intervals (CIs) were calculated for each gene to assess its association with GC risk. To assess the stability of the OR estimates, we performed non-parametric bootstrap resampling (1000 iterations) using the boot package in R, with bias-corrected accelerated (BCa) confidence intervals reported via the boot.ci function. The model’s discriminative performance was evaluated by the area under the receiver operating characteristic curve (AUC) in both the training set and an external validation set (GSE54129).

### 4.7. Diagnostic Performance Analysis of Hub Genes

Furthermore, to evaluate the prognostic relevance of the three hub genes, survival analysis was performed using the Kaplan–Meier plotter online tool (KM plotter, https://kmplot.com, accessed on 10 August 2025), which integrates gene expression and clinical follow-up data from a large cohort of 875 GC patients derived from GEO and TCGA databases [39]. For each hub gene, patients were divided into high-expression and low-expression groups using the platform’s default “best separation” cutoff strategy, which automatically selects the expression threshold that maximizes the log-rank test statistic between the two groups. Kaplan–Meier survival curves were generated, and log-rank tests were conducted to compare overall survival differences between the two groups with high and low expression of each hub gene. Hazard ratios (HRs) with 95% confidence intervals (CIs) and *p*-values were calculated using univariate Cox regression.

### 4.8. Single-Cell Expression and Distribution Analysis of Key Genes

The spatial expression and distribution of key genes across different cell types in GC and normal tissues were analyzed using the DIY expression profiling module of the scCancerExplorer database [40]. This investigation was conducted using the publicly available single-cell dataset GSE210347 from the NCBI GEO repository. The expression values provided by the database were normalized relative expression levels. In the violin plot, the y-axis represented the relative expression level.

### 4.9. Gene Set Enrichment Analysis (GSEA) of Hub Genes for BaP-Related GC Pathogenesis

To investigate the potential biological functions and pathways associated with the identified potential hub genes of BaP-related GC, single-gene Gene Set Enrichment Analysis (GSEA) was performed. For the single-gene GSEA, the “GSVA” package (version 1.52.3) in R was employed to calculate enrichment scores for pre-defined gene sets from the Molecular Signatures Database (MSigDB v2025.1.Hs) [41]. The statistical significance of the enrichment results was assessed using the *t*-test method, with a *p*-value < 0.05 considered indicative of a significant association.

### 4.10. Molecular Docking

To determine if BaP can bind to the methyltransferase enzymes that caused abnormal expression of hub genes of BaP-related GC, molecular docking was carried out. The docking experiments were performed using MOE version 2022.02, targeting BaP against DNMT1 and DNMT3A enzymes. Crystal structures of DNMT1 (PDB code: 4WXX) and DNMT3A (PDB code: 6BRR) were obtained from the RCSB Protein Data Bank [42]. Initially, all crystallographic water molecules were removed, and hydrogen atoms were added according to the AMBER99 force field parameters. Energy minimization was then conducted with an RMSD gradient convergence criterion of 0.05 kcal/mol·Å. Protonation of the methyltransferases was performed prior to a second round of minimization under the same force field and convergence threshold. The triangle matcher algorithm was employed to find the best binding poses. Ultimately, the binding affinities and interactions between BaP and the target enzymes were evaluated using the London dG rescoring method integrated with force field scoring.

### 4.11. Molecular Dynamics Simulation

To further analyze the receptor–ligand complex exhibiting the strongest binding from docking results, molecular dynamics simulations were conducted utilizing Gromacs 2023.3 software [43]. The starting configuration was derived from the docking output, followed by energy minimization. The system, prepared with the AMBER ff19SB and GAFF2 force fields, was solvated in a water box with ions to mimic physiological conditions. Following energy minimization and standard NVT/NPT equilibration, the production simulation was conducted at 298.15 K and 1 bar, employing a 2 fs timestep. Finally, a 100-nanosecond production simulation was performed to assess the dynamic stability and behavior of the complex.

### 4.12. Drug Enrichment Analysis

To identify potential therapeutic agents targeting the key genes associated with BaP-related GC, we performed a drug–gene enrichment analysis based on the DSigDB 1.0 database [44]. The analysis was conducted using the enricher function from the clusterProfiler package, with the drug–gene interaction data from DSigDB serving as the reference set. A significance threshold of *p*-value < 0.05 was applied. The significantly enriched drugs were visualized through multiple methods including bar plots, dot plots, and network diagrams. Finally, candidate drugs most relevant to the key gene set and exhibiting the highest interaction scores were selected.

### 4.13. Cell Experiments

Human GC AGS cells (cat. no. TCH-C124) and human normal gastric epithelial GES-1 cells (cat. no. TCH-C410) were obtained from HaiXing Bio (Suzhou, China). They were cultured in Ham’s F12K medium (cat. no. GUMD-B307) and DMEM (cat. no. PYG0073), respectively, both supplemented with 10% fetal bovine serum and 1% penicillin–streptomycin. Cells were maintained at 37 °C in a 5% CO_2_ incubator (Thermo Fisher Scientific, Waltham, MA, USA). Cells in the logarithmic growth phase were seeded into 96-well plates (Corning Inc., Corning, NY, USA) at a density of 5 × 10^3^ cells per well. After 24 h of culture, cells were treated for 48 h with graded concentrations of 5-azacytidine (Proteintech Group, Inc. Rosemont, IL, USA, cat. no. CM01266; 0, 0.5, 1, 2.5, 5, 10, 20 μM) or oxaliplatin (Proteintech Group, Inc., Rosemont, IL, USA, cat. no. CM00422; 0, 1, 2.5, 5, 10, 20, 40, 200 μM). The CCK-8 assay was then performed, and the absorbance at 450 nm was measured using a microplate reader (Beijing Perlong New Technology Co., Ltd., Beijing, China). The IC_30_ and IC_50_ values were calculated, and the selectivity index (GES-1 IC_50_/AGS IC_50_) was determined. To compare the regulatory effects of single agent versus combined treatment on the DNMT3A–KCNE2 axis under comparable levels of low cytotoxicity, we treated AGS cells with single agents at their respective IC_30_ concentrations (5-azacytidine and oxaliplatin) or with the combination at the IC_20_ concentration of each agent (to keep total cytotoxicity approximately equivalent to the single agent IC_30_). For formal experiments, AGS cells were divided into four groups: control (DMSO), 5-azacytidine (AGS IC_30_), oxaliplatin (AGS IC_30_), and combination (oxaliplatin IC_20_ + 5-azacytidine IC_20_). After 48 h of treatment, cell viability was assessed using the CCK-8 assay. Meanwhile, cells from each group were collected, and total RNA was extracted using TRIzol reagent. After reverse transcription, quantitative real-time PCR was performed using the SYBR Green method. The relative expression levels of DNMT3A and KCNE2 were calculated using the 2^−^ΔΔCt method with GAPDH as the internal control. All experiments were independently repeated three times. Comparisons among multiple groups were performed using a one-way analysis of variance (ANOVA), and a *p*-value < 0.05 was considered statistically significant.

### 4.14. Protein Level Detection

AGS cells were treated as described in Section 4.13. After 48 h, cells were lysed in RIPA buffer (Boster Biological Technology Co., Ltd., Wuhan, China, cat. no. AR0102) supplemented with protease and phosphatase inhibitors. Protein concentration was determined using a BCA kit (Boster Biological Technology Co., Ltd., Wuhan, China, cat. no. AR0146).

For Western blot analysis, equal amounts of protein (17.5 μg per lane) were separated by 12% SDS-PAGE and transferred onto PVDF membranes (Merck, KGaA, Darmstadt, Germany, cat. no. ISEQ00010) at 400 mA for 30 min. Membranes were blocked with 5% non-fat milk in TBST for 2 h, then incubated overnight at 4 °C with primary antibodies: rabbit anti-KCNE2 (1:1000, cat. No. A9859, ABclonal, Wuhan, China) and mouse anti-GAPDH (1:20,000, cat. no. 60004-1-Ig, Proteintech Group, Inc., Rosemont, IL, USA). After washing, membranes were incubated with HRP-conjugated secondary antibodies (1:5000, cat. no. SA00001-2/SA00001-1, Proteintech Group, Inc., Rosemont, IL, USA) for 1.5 h. Protein bands were visualized using an enhanced chemiluminescence kit (Proteintech Group, Inc., Rosemont, IL, USA, cat. no. PK10003) and detected with a Tanon 5200 imaging system. Densitometric analysis was performed using ImageJ software (version 1.54g, National Institutes of Health, Bethesda, MD, USA), and KCNE2 protein expression was normalized to GAPDH as the loading control.

For DNMT3A quantification, a commercial ELISA kit (Human DNMT3A ELISA Kit, Proteintech Group, Inc., Rosemont, IL, USA, cat. no. KE00812) was used. Cell lysates were diluted 1:20 with Sample Diluent PT1, and the assay was performed according to the manufacturer’s instructions. DNMT3A concentration was calculated from a standard curve using four-parameter logistic regression. Total protein concentration was determined by a BCA assay, and DNMT3A levels were expressed as ng per mg total protein. All samples were measured in duplicate.

## 5. Conclusions

This study is the first to systematically investigate gastric cancer from the perspective of network toxicology of food contaminants. By integrating multidisciplinary approaches and starting from the foodborne carcinogen BaP, three core genes (KCNE2, SULF1, and TIMP1) were identified. A risk prediction model achieved an AUC of 0.988–0.998. Survival analysis confirmed their prognostic value (HRs: 1.24, 1.26, and 1.92, respectively). Single-cell and pathway analyses revealed that these genes regulate stroma–epithelial interactions and tumor-related pathways. Validation in specific cellular compartments of the tumor microenvironment confirmed that these genes mediate stromal–epithelial interactions and tumor progression. GSVA revealed that they regulate epigenetics, ECM remodeling, TGF-β signaling, and metabolic reprogramming. Mechanistically, the interaction suggests that BaP may function as a competitive inhibitor, potentially interfering with the binding of DNMT substrate. Drug enrichment and cellular validation demonstrated that the DNMT inhibitor 5-azacytidine downregulated DNMT3A, restored KCNE2 expression, and selectively inhibited gastric cancer cells (selectivity index 13.37). However, low-dose combination with oxaliplatin failed to enhance KCNE2 recovery, suggesting antagonistic transcriptional effects. In summary, KCNE2 is a novel protective target, and 5-azacytidine possesses therapeutic potential. This study provides a framework for drug repurposing based on environmental contaminant mechanisms and warrants further mechanistic and clinical translation studies.

## Figures and Tables

**Table 1:** Logistic regression analysis of hub genes for GC risk prediction.

Variable	B	SE	Z-Value	VIF	*p*-Value	OR	95% CI for OR	Boot_Mean OR	95% BCa CI for OR
KCNE2	−0.664	0.151	−4.396	1.016	<0.0001	0.515	0.374–0.682	0.502	0.338–0.778
SULF1	1.078	0.379	2.845	1.329	0.004	2.940	1.439–6.461	3.930	1.232–7.964
TIMP1	1.677	0.534	3.142	1.316	0.002	5.351	2.020–16.743	7.313	1.975–15.820
Constant	−22.652	5.375	−4.215		<0.0001				

B represents regression coefficient; SE represents standard error; Z-value represents the Wald test statistic; VIF represents degrees of freedom; *p*-value represents significance level; OR represents odds ratio.

## Data Availability

The data used to support the findings of this study are included within the article.

## Supplementary Materials

The following supporting information can be downloaded at: https://www.mdpi.com/article/10.3390/ph19071060/s1, Figure S1: RMSF of the DNMT1 amino acid residues. Figure S2: RMSF of the DNMT3A amino acid residues. Figure S3: Changes in intraprotein hydrogen bonds during the kinetic process of DNMT1 (A) and DNMT3A (B). Figure S4:The protein level of DNMT3A and KCNE2. Table S1: Top five significantly upregulated and downregulated pathways from GSVA analysis for each hub gene.
