# Supplementary material for: From Food Contaminant to Therapeutic Target: Identification of KCNE2 and 5-Azacytidine for Gastric Cancer via Multi-Omics, Machine Learning, and In Vitro Validation
Source: Pharmaceuticals (Basel). 2026 Jul 9;19(7):1060. doi: 10.3390/ph19071060 (PMC13416207; doi:10.3390/ph19071060)
Supplement: Supplementary file 1 [file pharmaceuticals-19-01060-s001.zip › pharmaceuticals-4284283-supplementary.pdf]

Table S1 Top five significantly upregulated and downregulated pathways from GSVA analysis for each hub gene

	Pathway	t	P value	Type
KEGG GSVA based KCNE2	KEGG_BLADDER_CANCER	-3.428	0.001	Down
	KEGG_NOTCH_SIGNALING_PATHWAY	-3.324	0.001	Down
	KEGG_AXON_GUIDANCE	-2.903	0.004	Down
	KEGG_FOLATE_BIOSYNTHESIS	-2.809	0.006	Down
	KEGG_SULFUR_METABOLISM	-2.723	0.007	Down
	KEGG_MATURITY_ONSET_DIABETES_OF_THE_YOUNG	-2.657	0.009	Down
	KEGG_BASAL_CELL_CARCINOMA	-2.462	0.015	Down
	KEGG_GLIOMA	-2.441	0.016	Down
	KEGG_GLYCOSAMINOGLYCAN_BIOSYNTHESIS_HEPARAN_SULFATE	-2.396	0.018	Down
	KEGG_MELANOMA	-2.249	0.026	Down
	KEGG_ASCORBATE_AND_ALDARATE_METABOLISM	3.559	<0.001	Up
	KEGG_VALINE_LEUCINE_AND_ISOLEUCINE_DEGRADATION	3.607	<0.001	Up
	KEGG_GLYCINE_SERINE_AND_THREONINE_METABOLISM	3.708	<0.001	Up
	KEGG_PHENYLALANINE_METABOLISM	3.764	<0.001	Up
	KEGG_ENDOCYTOSIS	3.768	<0.001	Up
	KEGG_FATTY_ACID_METABOLISM	3.865	<0.001	Up
	KEGG_ARGININE_AND_PROLINE_METABOLISM	3.965	<0.001	Up
	KEGG_GLYCOLYSIS_GLUCONEOGENESIS	4.036	<0.001	Up
	KEGG_HISTIDINE_METABOLISM	4.602	<0.001	Up
	KEGG_NITROGEN_METABOLISM	5.094	<0.001	Up
GO GSVA based KCNE2	GOBP_REVERSIBLE_DIFFERENTIATION	-5.564	<0.001	Down
	GOBP_NEGATIVE_REGULATION_OF_MUSCLE_CELL_DIFFERENTIATION	-5.546	<0.001	Down
	GOBP_PHENOTYPIC_SWITCHING	-5.528	<0.001	Down
	GOBP_ASCENDING_AORTA_DEVELOPMENT	-5.467	<0.001	Down
	GOCC_CONE_PHOTORECEPTOR_OUTER_SEGMENT	-5.408	<0.001	Down
	GOBP_ANATOMICAL_STRUCTURE_REGRESSION	-5.282	<0.001	Down
	GOBP_CELL_DIFFERENTIATION_INVOLVED_IN_PHENOTYPIC_SWITCHING	-5.130	<0.001	Down
	GOBP_REGULATION_OF_PHENOTYPIC_SWITCHING	-5.130	<0.001	Down
	GOBP_EMBRYONIC_PLACENTA_DEVELOPMENT	-5.122	<0.001	Down
	GOBP_NEGATIVE_REGULATION_OF_MYOTUBE_DIFFERENTIATION	-5.119	<0.001	Down
	GOMF_ENDONUCLEASE_ACTIVITY_ACTIVE_WITH_EITHER_RIBO_OR_DEOXYRIBONUCLEIC_ACIDS_AND_PRODUCING_3_PHOSPHOMONOESTERS	5.600	<0.001	Up
	GOBP_GLUCOSE_IMPORT_IN_RESPONSE_TO_INSULIN_STIMULUS	5.642	<0.001	Up
	GOMF_TRACE_AMINE_RECEPTOR_ACTIVITY	5.654	<0.001	Up

	GOBP_NEGATIVE_REGULATION_OF_VOLTAGE_GATED_POTASSIUM_CHANNEL_ACTIVITY	5.740	<0.001	Up
	GOBP_MEMBRANE_DOCKING	5.757	<0.001	Up
	GOBP_VESICLE_DOCKING_INVOLVED_IN_EXOCYTOSIS	5.770	<0.001	Up
	GOCC_CHROMAFFIN_GRANULE	6.099	<0.001	Up
	GOBP_VESICLE_DOCKING	6.233	<0.001	Up
	GOBP_REGULATION_OF_DELAYED_RECTIFIER_POTASSIUM_CHANNEL_ACTIVITY	6.801	<0.001	Up
	GOBP_NEGATIVE_REGULATION_OF_DELAYED_RECTIFIER_POTASSIUM_CHANNEL_ACTIVITY	7.140	<0.001	Up
	KEGG_VALINE_LEUCINE_AND_ISOLEUCINE_DEGRADATION	-3.995	<0.001	Down
	KEGG_AMINOACYL_TRNA_BIOSYNTHESIS	-3.855	<0.001	Down
	KEGG_PEROXISOME	-3.776	<0.001	Down
	KEGG_PURINE_METABOLISM	-3.733	<0.001	Down
	KEGG_SPLICEOSOME	-3.694	<0.001	Down
	KEGG_GLYCOSYLPHOSPHATIDYLINOSITOL_GPI_ANCHOR_BIOSYNTHESIS	-3.651	<0.001	Down
	KEGG_BASE_EXCISION_REPAIR	-3.587	<0.001	Down
	KEGG_NUCLEOTIDE_EXCISION_REPAIR	-3.582	<0.001	Down
	KEGG_DNA_REPLICATION	-3.509	0.001	Down
KEGG GSVA based SULF1	KEGG_RNA_DEGRADATION	-3.489	0.001	Down
	KEGG_PROSTATE_CANCER	3.548	0.001	Up
	KEGG_DILATED_CARDIOMYOPATHY	3.619	<0.001	Up
	KEGG_PATHOGENIC_ESCHERICHIA_COLI_INFECTION	3.842	<0.001	Up
	KEGG_HYPERTROPHIC_CARDIOMYOPATHY_HCM	3.905	<0.001	Up
	KEGG_ARRHYTHMOGENIC_RIGHT_VENTRICULAR_CARDIOMYOPATHY_ARVC	3.932	<0.001	Up
	KEGG_GLYCOSAMINOGLYCAN_BIOSYNTHESIS_CHONDROITIN_SULFATE	4.578	<0.001	Up
	KEGG_TGF_BETA_SIGNALING_PATHWAY	5.071	<0.001	Up
	KEGG_PATHWAYS_IN_CANCER	5.366	<0.001	Up
	KEGG_FOCAL_ADHESION	6.138	<0.001	Up
	KEGG_ECM_RECEPTOR_INTERACTION	7.631	<0.001	Up
	GOBP_G1_TO_G0_TRANSITION	-5.373	<0.001	Down
	GOCC_EUCHROMATIN	-5.029	<0.001	Down
	GOBP_DNA_METHYLATION_DEPENDENT_HETEROCHROMATIN_FORMATION	-4.994	<0.001	Down
GO GSVA based SULF1	GOMF_NEUROTRANSMITTER_TRANSMEMBRANE_TRANSPORTER_ACTIVITY	-4.923	<0.001	Down
	GOBP_MRNA_MODIFICATION	-4.910	<0.001	Down
	GOMF_ACETYLTRANSFERASE_ACTIVITY	-4.884	<0.001	Down
	GOBP_TETRAPYRROLE_BIOSYNTHETIC_PROCESS	-4.853	<0.001	Down
	GOMF_OXIDOREDUCTASE_ACTIVITY_ACTING_ON_THE_CH_	-4.849	<0.001	Down

	CH_GROUP_OF_DONORS_WITH_A_FLAVIN_AS_ACCEPTOR			
	GOMF_MAP_KINASE_KINASE_ACTIVITY	-4.833	<0.001	Down
	GOBP_MITOTIC_INTRA_S_DNA_DAMAGE_CHECKPOINT_SIGNALING	-4.826	<0.001	Down
	GOMF_COLLAGEN_BINDING	7.618	<0.001	Up
	GOBP_PEPTIDYL_LYSINE_OXIDATION	7.689	<0.001	Up
	GOMF_PROTEIN_LYSINE_6_OXIDASE_ACTIVITY	7.689	<0.001	Up
	GOBP_ENDODERM_FORMATION	7.798	<0.001	Up
	GOCC_LAMELLIPODIUM_MEMBRANE	7.919	<0.001	Up
	GOBP_NEGATIVE_REGULATION_OF_EXTRACELLULAR_MATRIX_ORGANIZATION	7.926	<0.001	Up
	GOMF_EXTRACELLULAR_MATRIX_BINDING	8.118	<0.001	Up
	GOBP_MELANOCYTE_PROLIFERATION	8.377	<0.001	Up
	GOBP_ENDODERMAL_CELL_DIFFERENTIATION	8.526	<0.001	Up
	GOBP_NEGATIVE_REGULATION_OF_EXTRACELLULAR_MATRIX_DISASSEMBLY	8.584	<0.001	Up
KEGG GSVA based TIMP1	KEGG_VALINE_LEUCINE_AND_ISOLEUCINE_DEGRADATION	-6.786	<0.001	Down
	KEGG_CITRATE_CYCLE_TCA_CYCLE	-6.103	<0.001	Down
	KEGG_PYRUVATE_METABOLISM	-5.299	<0.001	Down
	KEGG_GLYOXYLATE_AND_DICARBOXYLATE_METABOLISM	-4.850	<0.001	Down
	KEGG_PEROXISOME	-4.814	<0.001	Down
	KEGG_VALINE_LEUCINE_AND_ISOLEUCINE_BIOSYNTHESIS	-4.682	<0.001	Down
	KEGG_PROPANOATE_METABOLISM	-4.635	<0.001	Down
	KEGG_BUTANOATE_METABOLISM	-4.607	<0.001	Down
	KEGG_OLFACTORY_TRANSDUCTION	-4.347	<0.001	Down
	KEGG_HOMOLOGOUS_RECOMBINATION	-4.286	<0.001	Down
	KEGG_LEUKOCYTE_TRANSENDOTHELIAL_MIGRATION	4.179	<0.001	Up
	KEGG_MTOR_SIGNALING_PATHWAY	4.238	<0.001	Up
	KEGG_TAURINE_AND_HYPOTAURINE_METABOLISM	4.344	<0.001	Up
	KEGG_CYTOKINE_CYTOKINE_RECEPTOR_INTERACTION	4.468	<0.001	Up
	KEGG_PATHWAYS_IN_CANCER	4.796	<0.001	Up
	KEGG_TGF_BETA_SIGNALING_PATHWAY	5.025	<0.001	Up
	KEGG_FOCAL_ADHESION	5.405	<0.001	Up
	KEGG_GLYCOSAMINOGLYCAN_BIOSYNTHESIS_CHONDROITIN_SULFATE	6.609	<0.001	Up
	KEGG_ECM_RECEPTOR_INTERACTION	7.138	<0.001	Up
	KEGG_COMPLEMENT_AND_COAGULATION_CASCADES	7.429	<0.001	Up
GO GSVA based TIMP1	GOBP_LEUCINE_METABOLIC_PROCESS	-6.732	<0.001	Down
	GOBP_BRANCHED_CHAIN_AMINO_ACID_CATABOLIC_PROCESS	-6.635	<0.001	Down
	GOBP_LEUCINE_CATABOLIC_PROCESS	-6.539	<0.001	Down
	GOBP_BRANCHED_CHAIN_AMINO_ACID_METABOLIC_PROCESS	-6.198	<0.001	Down

GOMF_ACETYLTRANSFERASE_ACTIVITY	-5.994	<0.001	Down
GOBP_TRICARBOXYLIC_ACID_CYCLE	-5.855	<0.001	Down
GOBP_RESPONSE_TO_ALKALOID	-5.763	<0.001	Down
GOMF_PROTEIN_SERINE_THREONINE_TYROSINE_KINASE_ACTIVITY	-5.730	<0.001	Down
GOCC_MITOCHONDRIAL_MATRIX	-5.721	<0.001	Down
GOBP_VALINE_METABOLIC_PROCESS	-5.650	<0.001	Down
GOBP_ENDODERMAL_CELL_DIFFERENTIATION	8.562	<0.001	Up
GOBP_NEGATIVE_REGULATION_OF_COAGULATION	8.635	<0.001	Up
GOBP_NEGATIVE_REGULATION_OF_PLASMINOGEN_ACTIVATION	8.730	<0.001	Up
GOBP_POST_EMBRYONIC_ANIMAL_MORPHOGENESIS	8.868	<0.001	Up
GOBP_REGULATION_OF_EXTRACELLULAR_MATRIX_DISASSEMBLY	8.902	<0.001	Up
GOBP_POST_EMBRYONIC_EYE_MORPHOGENESIS	9.060	<0.001	Up
GOBP_COLLAGEN_BIOSYNTHETIC_PROCESS	9.209	<0.001	Up
GOCC_INTERPHOTORECEPTOR_MATRIX	9.367	<0.001	Up
GOBP_POST_EMBRYONIC_ANIMAL_ORGAN_MORPHOGENESIS	9.400	<0.001	Up
GOBP_REGULATION_OF_COLLAGEN_METABOLIC_PROCESS	9.402	<0.001	Up

P-values < 0.001 were reported as < 0.001; p-values between 0.001 and 0.009 were rounded to three decimal places.

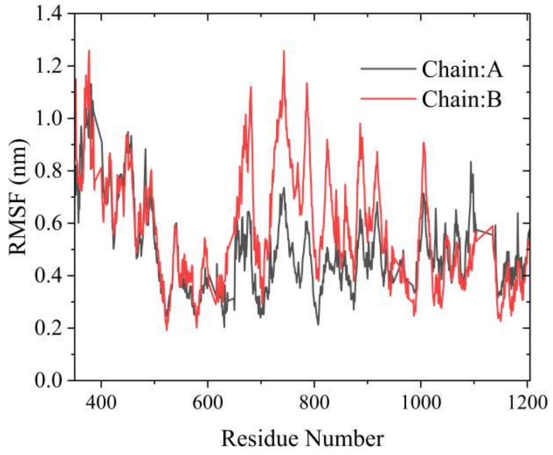

Figure S1. RMSF of the DNMT1 amino acid residues.

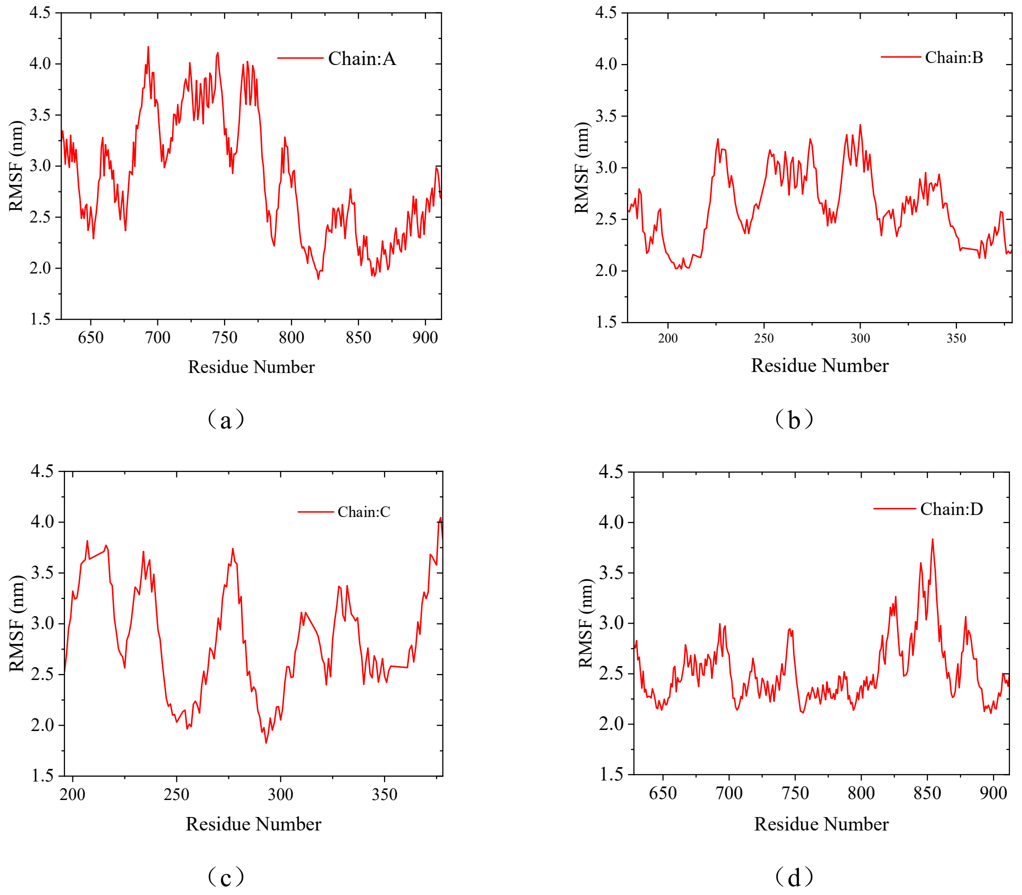

Figure S2. RMSF of the DNMT3A amino acid residues.

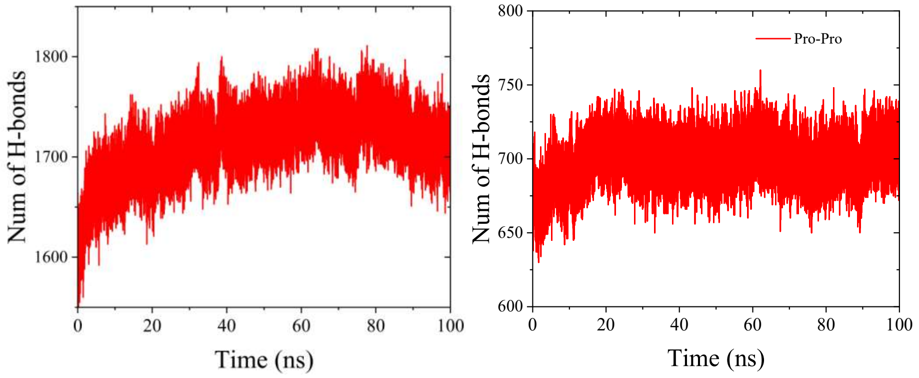

A: DNMT1 B: DNMT3A

Figure S3. Changes in intraprotein hydrogen bonds during the kinetic process of DNMT1 (A) and DNMT3A (B).

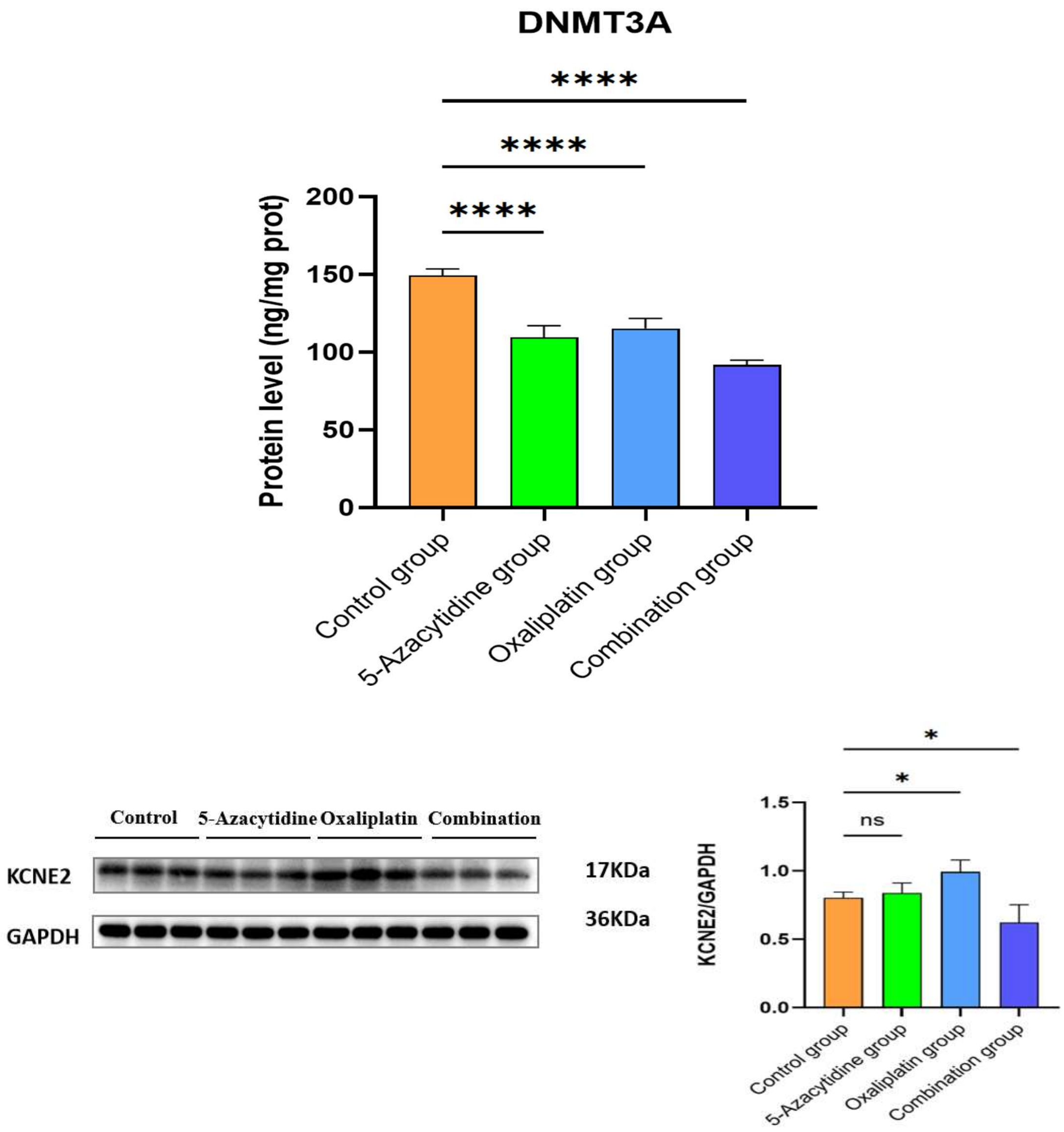

Figure S4. The protein level of DNMT3A and KCNE2. *$p<0.05$, $^{ns}p>0.05$, ****$p<0.0001$ compared with the control group
